# Predischarge Dysphagia Measured Using the Eating Assessment Tool-10 (EAT-10) and Its Association With 90-Day Aspiration Pneumonia and Hospital Readmission

**DOI:** 10.7759/cureus.93643

**Published:** 2025-10-01

**Authors:** Samraiz Nafees, Khalid Shahzad, Imad Sibhai, Yashar Mashayekhi, Sami Ullah Khan, Sana Omer Mian, Abra Zahid, Khaled Mohamed, Gyanendra K C, Muhammad Ibrar, Mustafa Al Hamdani

**Affiliations:** 1 Internal Medicine, Services Institute of Medical Sciences, Lahore, PAK; 2 Internal Medicine, King Edward Medical University, Lahore, PAK; 3 Internal Medicine, Smt. Nathiba Hargovandas Lakhmichand (NHL) Municipal Medical College, Ahmedabad, IND; 4 Medicine, University Hospitals of Leicester NHS Trust, Leicester, GBR; 5 General Medicine, Rawal Institute of Health Sciences, Islamabad, PAK; 6 Internal Medicine, Fatima Jinnah Medical College, Lahore, PAK; 7 Internal Medicine, Shaikh Khalifa Bin Zayed Al-Nahyan Medical and Dental College, Lahore, PAK; 8 Respiratory Medicine, Walsall Manor Hospital, Walsall, GBR; 9 Radiology, KIST Medical College & Teaching Hospital, Lalitpur, NPL; 10 Medicine, Mohammed Bin Rashid University of Medicine and Health Sciences, Dubai, ARE; 11 Pediatrics, Gulf Medical University, Ajman, ARE

**Keywords:** aspiration pneumonia, dysphagia, eat-10, hospital readmission, swallowing disorders

## Abstract

Background

Dysphagia is a swallowing disorder that may give rise to severe complications when not managed at the initial stages. Discharged patients with unresolved swallowing problems may become vulnerable to aspiration pneumonia and unexpected hospital readmissions. The purpose of this study was to establish the connection between predischarge dysphagia, measured with the Eating Assessment Tool-10 (EAT-10), and the incidence of aspiration pneumonia and 90-day hospital readmission.

Methods

The study was a prospective observational study conducted from January to June 2025 in hospitals in Lahore. The 511 clinically stable participants discharged were evaluated regarding dysphagia, demographic, and clinical information using the EAT-10. The incidence of aspiration pneumonia and readmission was established using follow-up at 90 days. Correlation tests, including the Mann-Whitney U test, the Kruskal-Wallis H test, the chi-square test, and logistic regression, were used to analyze the data.

Results

The significant scores on the EAT-10 were related to aspiration pneumonia (r = 0.245, p < 0.001) and 90-day readmission (r = 0.310, p < 0.001). Patients who scored high on the EAT-10 were more likely to develop pneumonia (p < 0.001) and readmission (p < 0.001). Other predictors included older age, comorbidities, a history of pneumonia, and an extended hospital stay. Logistic regression supported EAT-10 as a significant predictor of both pneumonia (OR = 1.03, p = 0.012) and readmission (OR = 1.01, p = 0.040).

Conclusions

Predischarge dysphagia, as measured by EAT-10, is strongly related to post-discharge complications such as aspiration pneumonia and readmission. Regular screening during discharge can help identify high-risk patients and enable timely interventions, thereby enhancing patient safety and outcomes.

## Introduction

Dysphagia is a condition characterized by a weakness of the swallowing apparatus, and it can lead to the penetration or aspiration of food or secretions into the airway. Normal swallowing is a multifaceted process that is safe, conveying food and liquids to the stomach in a secure manner. Dysphagia may occur as a result of oral, pharyngeal, or esophageal malfunctions, which require close consideration of particular physiologic or anatomic anomalies [1,2]. Dysphagia occurs in 10-33% of the elderly and is characterized by malnutrition, pneumonia, and a higher mortality rate, especially among stroke survivors and victims of neurological diseases [3].

Dysphagia is frequently associated with cardiac manifestations, including pain, heartburn, and regurgitation in elderly patients. It can lead to psychosocial problems, including mealtime anxiety and social withdrawal, which strongly affect quality of life [4]. Dysphagia occurs in approximately one-third of patients with acute stroke, particularly hemorrhagic stroke or massive middle cerebral artery stroke, and is a powerful predictor of three-month mortality and disability [5].

Community-acquired pneumonia is one of the significant health issues in the elderly, as it may be caused by oropharyngeal aspiration as a result of dysphagia and poor cough reflex secondary to neurologic factors. The dysphagia, especially in patients who have experienced a stroke, significantly predisposes them to pneumonia as opposed to those who do not have a problem in swallowing [6,7]. It is also one of the significant risk factors of aspiration pneumonia and five-year mortality in patients with cerebral hemorrhage [8].

Notably, the definition of aspiration pneumonia itself remains a matter of debate, with recent reviews and international guidelines emphasizing its complex epidemiology, diagnostic variability, and evolving management approaches [9,10].

Dysphagia in acute geriatric patients relates to increased 30- and 90-day mortality and prolonged hospitalization, regardless of stroke, cancer, or neurodegenerative disease [11]. It is also associated with prolonged stays, elevated costs, and increased 30-day readmission rates, which underscores the importance of early detection and intervention [12].

Dysphagia is commonly underdiagnosed, especially in frail, neurological, or head and neck patients, where it causes complications like dehydration and aspiration pneumonia. Eating Assessment Tool-10 (EAT-10) is a valid and reliable self-administered diagnostic tool. A cutoff score of 3 is recommended for more effective detection of individuals at risk [13,14].

The study aims to determine the correlation between predischarge dysphagia measured with EAT-10 and the occurrence of aspiration pneumonia and 90-day hospital readmission. With the help of this association, we will be able to underline how regular screening of dysphagia contributes not only to the identification of high-risk patients but also to the creation of particular interventions aimed at minimizing preventable post-discharge complications.

Study rationale

Dysphagia is a condition that may persist even after a patient is medically fit to be discharged. If not noted, it may cause complications like aspiration pneumonia and unplanned readmissions. The monitoring tools, including the EAT-10, can enable a medical practitioner to quickly identify issues with swallowing and implement preventive measures, such as dietary changes or home-based treatments. Even though previous studies have been conducted on the relationship between predischarge dysphagia and post-discharge complications in other populations, the relationship between the two phenomena has not been comprehensively examined in the Pakistani healthcare context.

The importance of understanding the effects of predischarge dysphagia on 90-day readmissions and aspiration pneumonia is fundamental because the healthcare resources, patient population, and discharge activities in Pakistan might not be similar to those in other countries analyzed. Local research on this association could be implemented to identify at-risk patients more efficiently, inform discharge planning, and tailor interventions to the specific needs of the Pakistani population, thereby preventing the emergence of complications that can be avoided and enhancing patient safety.

Study goals

The primary objective of this study is to investigate the relationship between predischarge dysphagia, as assessed by the EAT-10 tool, and the incidence of aspiration pneumonia within 90 days after hospital discharge. The study will also attempt to determine whether predischarge dysphagia is related to unplanned rehospitalization during the same period. In addition to this, the study will also focus on exploring the prevalence and severity rates of dysphagia among the hospitalized patients before discharge and the means by which the two can influence the outcomes of the discharge. This study will be insightful as it will identify high-risk patients, inform clinical decision-making, enhance discharge planning, and implement early interventions to prevent aspiration events and avoidable readmission rates. Ultimately, the research will contribute to the existing evidence on improving patient safety and continuity of care within local healthcare settings.

## Materials and methods

Methods and approach

The correlation between predischarge dysphagia and the incidence of aspiration pneumonia and 90-day readmission was identified using a prospective observational study design. The patients were selected from the inpatient wards of Lahore hospitals (both public and private), as they have diverse backgrounds in terms of socioeconomic status and cultural backgrounds.

To gather the data, a structured questionnaire was used, including the EAT-10 tool to evaluate the issue regarding swallowing before discharge. The identified patients who were clinically stable were approached by trained research assistants who had left the hospital and explained the purpose and methods of the study to them. They had provided written or oral informed consent. The questionnaires were administered either as self-administered questionnaires or with the assistance of a research team member, depending on the participants’ literacy levels. The method helped ensure accurate data collection, as well as culturally competent and respectful communication within the local hospital environment.

Recruitment and sample details

To estimate the sample size, a formula from the World Health Organization was employed, using a 95% CI with a 5% margin of error and assuming a population proportion of 0.5, as the maximum variability was determined [15]. After these parameters, the sample size of 384 people was obtained as the minimum required sample size. The research methods aimed to approach 520 patients to participate in the study, gathering relevant data and handling nonresponses or incomplete data.

The study invited all eligible inpatients who were clinically stable for discharge to participate, using a nonrandom convenience sampling method. Among the overall number approached, only those who answered the EAT-10 questionnaire and submitted the corresponding clinical data were included in the final analysis, resulting in a sample of 511 patients. The sample size also exceeds the calculated minimum, which is sufficient to achieve the statistical power needed to answer the question of the association between predischarge dysphagia, 90-day aspiration pneumonia, and hospital readmissions, as well as to conduct meaningful subgroup analysis.

Eligibility criteria

The population consisted of adult patients aged 18 years and above who were admitted to the hospital and had a planned discharge and were considered clinically stable. Participants were able to complete the EAT-10 questionnaire independently or with the assistance of a research team member. Also, the patients had to be willing to undergo follow-up evaluations for a period of 90 days after discharge.

Patients who had undergone tracheostomy, gastrostomy, or any other artificial feeding route to the extent of a hole in the examination of swallowing were excluded. Those who were severely cognitively impaired or delirious, or who could not answer the questionnaire because of some communication barrier, were also disqualified. In addition, patients discharged against medical advice, those discharged to other care facilities where follow-up was not available, and those with an estimated life expectancy of less than 90 days were excluded from the study.

Instruments and measures

A structured questionnaire was designed to collect and assess comprehensive data pertinent to this research. The questionnaire consisted of three essential sections: demographics, the EAT-10 to evaluate post-discharge and predischarge dysphagia, and a 90-day follow-up to assess outcomes after discharge. The questionnaire’s primary goal was to evaluate the existence and severity of swallowing issues on discharge and to determine their relationship to the occurrence of additional aspiration pneumonia and hospital readmission. All instruments were given in their original English format. No cultural or language adaptation was done.

Demographic information

The first section of the questionnaire involved an in-depth collection of demographic and clinical data to facilitate subgroup analysis to determine the relationship between patient characteristics and dysphagia. The variables collected included age, gender, marital status, educational level, occupation, and whether the individual lived in an urban or rural area. Clinical data consisted of the primary diagnosis, comorbidities, hospital stay, and a history of pneumonia or respiratory issues. This data contributed to the contextualization of the patient’s risk profile and the identification of additional factors that could impact post-discharge outcomes.

EAT-10

EAT-10 was created by Belafsky et al. in 2008 as a patient-reported, simple screening tool for dysphagia. It consists of 10 questions that assess the difficulty in swallowing and its impact on daily activities. All items will be rated on a 5-point Likert scale, with 0 (no problem) at one end and 4 (severe problem) at the other, totaling a maximum possible score of 0-40. The greater the scores, the worse the swallowing problems. The instrument has been demonstrated to have high internal consistency, as indicated by a Cronbach’s alpha of 0.96. It is thus a valid predictive tool for patients who may be at risk of aspiration and other dysphagia-related complications [16]. The study used EAT-10 because it screened predischarge dysphagia, which is short-lived, easy to administer, and valid. In this study, patients with an EAT-10 score of ≥3 were classified as having dysphagia, consistent with published validation studies. We did not perform additional instrumental diagnostic tests such as videofluoroscopic swallowing study (VFSS) or fiberoptic endoscopic evaluation of swallowing (FEES) to establish the diagnosis. The underlying etiologies of dysphagia (e.g., stroke, neurological disease, and head and neck pathology) were not stratified separately; all eligible patients were screened uniformly with EAT-10 irrespective of primary diagnosis. Furthermore, no formal grading of dysphagia severity was conducted beyond the total EAT-10 score, which functioned as the screening tool. Formal permission for the use of the EAT-10 was obtained from Mapi Research Trust (Special Terms No. 121857).

Ninety-day follow-up section

The third part involved post-discharge outcomes within 90 days. Patients were asked about whether they had been diagnosed with pneumonia after discharge and whether they had been rehospitalized. If patients reported such events, these were further verified through available hospital records or discharge summaries. The diagnosis of aspiration pneumonia was determined by the treating physicians as documented in medical charts, and the research team only extracted this information without making independent diagnostic judgments. These questions and confirmations were crucial for gathering reliable information to assess the clinical implications of predischarge dysphagia and to establish potential relationships between EAT-10 scores and adverse outcomes.

Data collection process

The eligible patients were approached upon their hospitalization when they were stable and ready to be discharged. The study’s purpose and procedures were clearly outlined, and informed consent was obtained either orally or in writing. A structured questionnaire consisting of demographic and EAT-10 was administered by trained research assistants who used the questionnaire to assess predischarge dysphagia. The data collection time was within six months, from January 2025 to June 2025.

The questionnaire was either self-administered or administered with the assistance of the research team, depending on the patient’s literacy level and their preference. The follow-up data were obtained through telephone calls made by the research team or by visiting patients who returned to the hospital for routine care or to address complications. This methodology enabled a detailed follow-up of post-discharge results, allowing for flexibility to support patient preferences and availability. All data were systematically recorded to easily analyze the relationship between predischarge dysphagia and the resultant clinical outcome.

Data analysis strategy

IBM SPSS Statistics for Windows, Version 26.0 (Released 2018; IBM Corp., Armonk, NY, USA) was used to analyze data. The demographic and clinical characteristics of the 511 participants were summarized using descriptive statistics (frequencies and percentages). The scores of the EAT-10 were evaluated for normality using a detrended normal Q-Q plot. The data were not normally distributed; therefore, nonparametric tests were used. The Spearman correlation analysis was used to evaluate the relationships between EAT-10 scores, aspiration pneumonia, and 90-day hospital readmission. The Mann-Whitney U test was used to compare group differences in EAT-10 scores based on the diagnosis of pneumonia and hospital readmission. The Kruskal-Wallis H test was used to compare EAT-10 scores and aspiration pneumonia in different age groups. Binary logistic regression analysis was performed to identify predictors of 90-day aspiration pneumonia and hospital readmission, using EAT-10 scores and clinical variables (cause of admission, comorbidities, history of pneumonia, and length of hospital stay) as predictors. Finally, the chi-square test was employed to identify the correlation between pneumonia diagnosis and 90-day hospital readmission. All statistical tests were two-tailed, with statistical significance being p < 0.05.

Research ethics and compliance

This research was conducted in accordance with the principles outlined in the Declaration of Helsinki regarding ethical considerations. The Shaikh Khalifa Bin Zayed Al-Nahyan Medical and Dental College (SKZMDC/IRB/25/013) provided ethical approval, and data collection was subsequently initiated. A clear description of the study’s purpose, procedures, risks, and benefits was provided to all participants. Informed consent was obtained from all subjects before their involvement, provided both in written and verbal form. The privacy and confidentiality of the study participants were highly maintained in the study. Data were coded and stored in a secure location, with access restricted to the research team only. The patients were informed that they had the right to withdraw from the study without affecting their ongoing medical treatment. The ethical processes were employed to ensure that all aspects of the study were conducted in a responsible manner that did not infringe upon the rights and well-being of the study participants.

## Results

Table 1 presents the demographic and clinical features of the study participants (N = 511). Most of the participants were aged ≥65 years (231, 45%), followed by 60-64 years (100, 19%), 45-59 years (80, 16%), 30-44 years (60, 12%), and 18-29 years (40, 8%). Males (270, 53%) were marginally higher than females (241, 47%). Almost half had only primary school education (234, 46%), secondary education (131, 26%), no formal education (79, 15%), higher secondary education (56, 11%), and graduate or higher education (11, 2%). The majority of participants were married (176, 34%), widowed (163, 32%), single (114, 22%), or divorced or separated (58, 12%). In terms of occupation, 168 (33%) were not employed, 155 (30%) were retired, 99 (19%) were employed, 61 (12%) were students, and 28 (6%) were homemakers. Head and neck cancer (179, 35%), stroke (139, 27%), and post-surgical conditions (121, 24%) were the primary causes of admission, and there were fewer cases of neurological disorders (50, 10%) or other causes (22, 4%). On discharge, 187 (37%) were on a modified diet, 160 (31%) were on tube feeding, 112 (22%) were placed on a regular oral diet, and 52 (10%) remained nil per os. Heart disease (192, 38%), chronic lung disease (119, 23%), diabetes (105, 20%), and chronic kidney disease (48, 9%) were the most common comorbidities, while only 3 (1%) did not report any comorbidities. Regarding smoking, 223 (44%) were former smokers, 155 (30%) never smoked, and 133 (26%) were current smokers. Reported pneumonia in the previous year was 289 (57%) participants. Hospital length of stay was less than five days (252, 49%), greater than five days (213, 42%), and greater than 10 days (46, 9%).

**Table 1:** Demographic characteristics of participants (N = 511) Values are presented as N (%). COPD, chronic obstructive pulmonary disease; NG, nasogastric; NPO, nil per os (nothing by mouth); PEG, percutaneous endoscopic gastrostomy

Variable	f (N)	%
Age		
18-29 years	40	8
30-44 years	60	12
45-59 years	80	16
60-64 years	100	19
65 years and above	231	45
Gender		
Male	270	53
Female	241	47
Educational level		
No formal education	79	15
Primary school (up to grade 5)	234	46
Secondary (grades 6-10)	131	26
Higher secondary (grades 11-12)	56	11
Graduate or above	11	2
Marital status		
Single	114	22
Married	176	34
Widowed	163	32
Divorced/separated	58	12
Occupation		
Student	61	12
Employed	99	19
Unemployed	168	33
Retired	155	30
Homemaker	28	6
Cause of admission		
Stroke	139	27
Head and neck cancer	179	35
Post-surgery (general/ENT/other)	121	24
Neurological disorder (e.g., Parkinson’s and dementia)	50	10
Other	22	4
Feeding status at discharge		
Normal oral diet	112	22
Modified diet (soft/thickened liquids)	187	37
Tube feeding (NG/PEG)	160	31
NPO	52	10
Comorbidities		
Hypertension	29	6
Diabetes	105	20
Heart disease	192	38
Chronic lung disease (COPD/asthma)	119	23
Chronic kidney disease	48	9
Cancer (other than current diagnosis)	15	3
None	3	1
Smoking status		
Never smoked	155	30
Former smoker	223	44
Current smoker	133	26
History of pneumonia in the past 12 months		
No	222	43
Yes	289	57
Length of hospital stay		
Less than five days	252	49
Five to 10 days	213	42
More than 10 days	46	9

Table 2 demonstrates that dysphagia severity, as measured by the EAT-10, had a significant positive correlation with both aspiration pneumonia (r = 0.245, p < 0.001) and 90-day hospital readmission (r = 0.310, p < 0.001). Also, hospital readmission was strongly correlated with aspiration pneumonia (ρ = 0.278, p < 0.001). These findings indicate that the more severe the dysphagia, the more likely one is to acquire aspiration pneumonia and be readmitted to the hospital. All the correlations were significant at p < 0.01.

**Table 2:** Spearman’s correlations between dysphagia severity, aspiration pneumonia, and hospital readmission within 90 days (N = 511) Values represent Spearman’s correlation coefficients (r) between continuous variables. p < 0.01 (two-tailed) was considered statistically significant and is denoted with double asterisks (^**^). EAT-10, Eating Assessment Tool-10

Variables	EAT-10	Aspiration pneumonia (within 90 days)	Hospital readmission (within 90 days)
EAT-10	-	ρ = 0.245, p < 0.001^**^	ρ = 0.310, p < 0.001^**^
Aspiration pneumonia (within 90 days)	-	-	ρ = 0.278, p < 0.001^**^
Hospital readmission (within 90 days)	-	-	-

Table 3 shows that participants who developed pneumonia 90 days after discharge had a significantly higher EAT-10 score (mean rank = 275.00) than those who did not (mean rank = 190.45). The Mann-Whitney U test demonstrated a statistically significant difference (U = 15232.00, Z = -5.41, p < 0.001), indicating that dysphagia severity is more strongly related to the probability of post-discharge pneumonia.

**Table 3:** Comparison of EAT-10 scores by pneumonia diagnosis status within 90 days after hospital discharge (N = 511) N = 511 (yes = 396, 78%; no = 115, 22%) The Mann-Whitney U test was used for all comparisons. p-Values marked with ^**^ indicate statistical significance at p < 0.01^**^. EAT-10, Eating Assessment Tool-10

Variable	Since your hospital discharge, have you been diagnosed with pneumonia?	N	Mean rank	Sum of ranks
EAT-10	No	115	190.45	21,902.00
	Yes	396	275	108,914.00
	Total	511	-	-
Test statistics	U	W	Z	p
EAT-10	15,232.00	21,902.00	-5.41	<0.001^**^

Table 4 shows that readmission within 90 days after discharge had much higher EAT-10 scores (mean rank = 274.04) than those who did not have readmission (mean rank = 180.25). This difference was statistically significant, as evidenced by the Mann-Whitney U test (U = 12814.00, Z = -5.65, p < 0.001), indicating that a higher level of dysphagia is strongly associated with an increased risk of 90-day hospital readmission.

**Table 4:** Comparison of EAT-10 scores by 90-day hospital readmission status after discharge (N = 511) N = 511 (yes = 413, 81%; no = 98, 19%) The Mann-Whitney U test was used for all comparisons. p-Values marked with ^**^ indicate statistical significance at p < 0.01^**^. EAT-10, Eating Assessment Tool-10

Variable	Have you been readmitted to the hospital within 90 days after discharge?	N	Mean rank	Sum of ranks
EAT-10	No	98	180.25	17,665.00
	Yes	413	274.04	113,151.00
	Total	511	-	-
Test statistics	U	W	Z	p
EAT-10	12,814.00	17,665.00	-5.65	<0.001^**^

Table 5 indicates that EAT-10 scores and the incidence of aspiration pneumonia improved gradually with age over the 90 days. The EAT-10 mean rank (310.75) and aspiration pneumonia (315.20) were highest in participants aged 65 years and above and lowest in the youngest group (18-29 years, 180.25 and 175.40, respectively). The Kruskal-Wallis H tests indicated that the differences by age were significant in both dysphagia severity (χ²(4) = 32.45, p < 0.001) and aspiration pneumonia (χ²(4) = 38.62, p < 0.001), indicating that older age is associated with increased dysphagia levels and increased risk of post-discharge aspiration pneumonia.

**Table 5:** Comparison of EAT-10 and aspiration pneumonia within 90 days across age groups (N = 511) Percentages are based on the total (N = 511). Values are mean ranks from Kruskal-Wallis H tests. Overall test statistics are reported. Total EAT (χ² (4) = 32.45, p = < 0.001**) and Total AP (χ² (4) = 38.62, p = < 0.001^**^); significance levels: p < 0.01^**^. EAT-10, Eating Assessment Tool-10

Age group	N (%)	Mean rank (Total_EAT)	Mean rank (Total_AP)
18-29 years	40 (8%)	180.25	175.4
30-44 years	60 (12%)	200.15	195.75
45-59 years	80 (16%)	240.35	235.1
60-64 years	100 (19%)	290.55	285.8
65 years & above	231 (45%)	310.75	315.2
Total	511	-	-
Test statistics	-	χ² (df = 4)	p
Total EAT	-	32.45	<0.001^**^
Total AP	-	38.62	<0.001^**^

Table 6 indicates that the predischarge level of EAT-10 was a significant predictor of 90-day aspiration pneumonia (OR = 1.03, p = 0.012) and hospital readmission (OR = 1.01, p = 0.040) after controlling for clinical factors. Additional significant predictors of aspiration pneumonia were cause of admission (OR = 1.04, p = 0.004), comorbidities (OR = 1.08, p = 0.002), history of pneumonia in the previous 12 months (OR = 1.03, p = 0.005), and length of hospital stay (OR = 2.50, p < 0.001). Equally, cause of admission (OR = 1.04, p = 0.009), comorbidities (OR = 1.07, p = 0.001), history of pneumonia (OR = 1.03, p = 0.007), and length of stay (OR = 1.02, p = 0.004) were significantly associated with hospital readmission. These findings show that the severity of dysphagia and some clinical factors are independent risk factors for post-discharge aspiration pneumonia and readmission within 90 days. Overall, these findings confirm that dysphagia severity (EAT-10 score) and several clinical factors were independent predictors of post-discharge aspiration pneumonia and hospital readmission.

**Table 6:** Logistic regression to predict 90-day aspiration pneumonia and readmission from predischarge clinical and EAT-10 factors (N = 511) Dependent variables were (a) pneumonia diagnosis within 90 days post-discharge and (b) hospital readmission within 90 days post-discharge. p < 0.05^*^, p < 0.01^**^, N = 511 EAT-10, Eating Assessment Tool-10; LL, lower limit; UL, upper limit

Outcome variable	Predictor	B	SE	Wald	p	OR	95% CI LL	95% CI UL
Aspiration pneumonia (within 90 days)	Constant	-2.36	0.61	14.98	<0.001^**^	^-^	-	-
	EAT-10 score	0.03	0.01	6.35	0.012^**^	1.03	1.01	1.05
	Cause of admission	0.04	0.01	8.42	0.004^**^	1.04	1.01	1.07
	Comorbidities	0.08	0.03	9.3	0.002^**^	1.08	1.03	1.14
	History of pneumonia (past 12 months)	0.03	0.01	8.01	0.005^**^	1.03	1.01	1.06
	Length of stay	0.92	0.15	37.5	<0.001^**^	2.5	1.87	3.35
Hospital readmission (within 90 days)	Constant	-1.98	0.52	14.64	<0.001^**^	-	-	-
	EAT-10 score	0.01	0.01	4.25	0.040^*^	1.01	1	1.03
	Cause of admission	0.04	0.02	6.8	0.009^**^	1.04	1.01	1.07
	Comorbidities	0.07	0.02	12.25	0.001^**^	1.07	1.03	1.11
	History of pneumonia (past 12 months)	0.03	0.01	7.29	0.007^**^	1.03	1.01	1.05
	Length of stay	0.02	0.01	8.51	0.004^**^	1.02	1.01	1.03

Table 7 shows a strong correlation between the diagnosis of pneumonia and readmission within 90 days of discharge. Among participants with pneumonia, 353 (69%) were readmitted within 90 days, compared to 60 (18%) in those without pneumonia. This association was statistically significant (χ²(1, N = 511) = 78.57, p < 0.001), indicating that post-discharge pneumonia significantly increases the likelihood of hospital readmission.

**Table 7:** Association between pneumonia diagnosis within 90 days of discharge and hospital readmission (N = 511) Percentages are based on the total sample (N = 511). The chi-square test revealed a significant association between pneumonia diagnosis within 90 days after discharge and hospital readmission. χ² (1, N = 511) = 78.57, p < 0.001^**^ EAT-10, Eating Assessment Tool-10

Pneumonia diagnosis within 90 days after discharge	Hospital readmission within 90 days (No, N (%))	Yes, N (%)	Total
No	55 (11%)	60 (18%)	115 (23%)
Yes	43 (8%)	353 (69%)	396 (77%)
Total	98 (19%)	413 (81%)	511 (100%)
Chi-square tests	χ²	df	p
Pearson chi-square	78.57	1	<0.001^**^
Continuity correction (2 × 2)	76.21	1	<0.001^**^

## Discussion

This study explored the relationship between predischarge dysphagia, measured using the EAT-10 tool, and the occurrence of aspiration pneumonia and hospital readmissions within 90 days of discharge. In our study, it was established that an increased EAT-10 score was strongly correlated with the onset of aspiration pneumonia within 90 days. Likewise, a previous study also found dysphagia as a significant predictor of pneumonia, thus adding to the correlation between impaired swallowing and the risk of pneumonia [17]. Our analysis revealed that higher EAT-10 scores were strongly associated with 90-day readmission. Equally, another nationwide study indicated that dysphagia is also another independent predictor of higher readmission rates, which underpins the significance of the swallowing impairment in hospital readmission [12]. In our research, it was evident that there is a strong correlation between aspiration pneumonia and a higher rate of hospital readmission within 90 days. Consistently, past research studies have also noted increased readmission rates in patients with aspiration pneumonia compared to those with community-acquired pneumonia, which further reiterates the increased rehospitalization risk associated with the condition [18].

Our analysis showed that patients with pneumonia in 90 days had a significantly higher EAT-10 score, which corresponds to worse dysphagia. On the same note, a previous study of inpatients with aspiration pneumonia indicated that dysphagia was very common, which affirms that swallowing disability is a significant cause of pneumonia irrespective of neurologic status [19].

In our research, those with higher EAT-10 scores were significantly correlated to a greater risk of 90-day hospital readmissions, highlighting the clinical and economic cost of dysphagia. Consistently, a recent study involving post-stroke patients showed severe dysphagia to independently predict increased acute hospitalization expenses, further exemplifying its role in influencing healthcare use [12,20].

Our results indicated that patients with older ages had substantially higher EAT-10 scores, which showed more severe dysphagia. It is in line with a meta-analysis, which found advanced age as a negative prognostic variable in terms of post-stroke dysphagia recovery [21]. Our findings indicated that aspiration pneumonia was much more common in older patients, especially in patients over the age of 65 years. This is in line with the prior literature concerning community-acquired pneumonia, which repeatedly points to the higher risk and poor prognosis of the older groups [22].

We have found that the EAT-10 score is positively correlated with an increased risk of pneumonia among older adults, which is consistent with the observation that muscle weakness in the aspiration and swallowing areas is a factor contributing to pneumonia in the elderly population [23]. The findings that the causes of admissions, along with the existence of several comorbidities, were also associated with increased risks of pneumonia reinforce the preexisting body of evidence. Notably, a cohort study supported by VFSS revealed that factors such as chronic obstructive pulmonary disease, tracheotomy, and hypertension were highly predictive of pneumonia, underscoring the importance of underlying health conditions in such predictions [17]. Our findings indicated that patients who had a history of recent pneumonia were at risk of developing recurrence. This confirms previously established facts that a history of pneumonia is a powerful predictor of future recurrence, which explains why it is essential to determine whether a patient is at risk of recurrence [24]. The findings of the study demonstrated that significantly more extended hospitalization is a predictor of the development of aspiration pneumonia, and it is associated with higher severity of illness and connected with high post-discharge morbidity and readmission [25].

A higher EAT-10 score was also marginally more likely to correlate with readmission at 90 days in our study. This is in line with the available literature, which reported that more severe dysphagia has a minor impact on elevating short-term readmission because there is a need to recognize and manage swallowing difficulty promptly to restrict hospital readmission [12]. We also find that the symptoms of predischarge dysphagia were predictive of 90-day readmission, which aligns with earlier analyses indicating that the etiology of the original hospital stay is a strong predictor of readmission in the short term. As in previous reports, specific admission diagnoses were found to have a higher readmission rate, underscoring the importance of considering underlying conditions during the hospital stay [26]. Comorbidities increase the risk of pneumonia, and our findings are consistent with earlier studies, including large-scale reviews on chronic respiratory diseases. These reviews also indicate that several underlying conditions are significant contributors to the risk of pneumonia. This suggests that comorbidities should be wisely managed to prevent pneumonia [27]. Our finding that a history of pneumonia is a risk factor for readmission is not new, as previous studies have also demonstrated that recent hospitalization and a history of prior episodes of illness are factors that predispose patients to repeat admissions in aspiration pneumonia cohorts [18]. Our findings, that more extended hospital stays are associated with a higher readmission risk, are in line with prior results, where more extended initial hospitalization was also associated with a higher readmission risk, presumably due to severity or complications during the initial stay [28].

We found that patients who had pneumonia within 90 days post-discharge were more likely to be readmitted significantly, which is consistent with the prior knowledge that post-pneumonia patients, particularly older adults and those with comorbidities, are at a greater risk of hospital readmission. This highlights the importance of strict follow-up and preventive measures after discharge [25].

Overall, these results highlight the severity of dysphagia, old age, comorbid conditions, pneumonia in the past, and extended hospital stay as the main risk factors that contribute to post-discharge pneumonia and hospital readmission. Prompt detection and intervention of swallowing disorders, close attention to at-risk patients, and active measures to control comorbidities can decrease morbidity, readmission, and medical bills.

Although this study highlights the predictive value of the EAT-10, it is essential to acknowledge its limitations and the debate surrounding its feasibility as a standalone tool. A recent systematic review and meta-analysis confirmed that subjective questionnaires, such as the EAT-10, may not always align with instrumental assessments, like videofluoroscopy, underscoring the need for cautious interpretation [29]. Moreover, the utility of EAT-10 may vary depending on the clinical condition; it is effective in detecting dysphagia and aspiration risk in early and moderate Alzheimer’s disease, but objective assessments, such as VFSS, remain essential in advanced stages [30]. In contrast, in Parkinson’s disease, EAT-10 is less reliable in detecting penetration and aspiration events [31]. Additionally, the very definition of aspiration pneumonia remains debated, with recent reviews and guidelines emphasizing variability in diagnostic criteria and evolving approaches to management [9,10]. These considerations suggest that while EAT-10 is a practical and valuable screening tool, its role should be considered complementary to objective diagnostic methods and disease-specific factors.

Study constraints

Several limitations should be considered. To start with, the research employed a convenience sampling method, which may result in selection bias and constraints in the generalizability of the findings to the remaining hospitals in the sample. Second, the diagnosis of aspiration pneumonia was made based on the patient’s report and clinical records, rather than on usual imaging or instrumental swallowing tests, which may result in misclassification. Third, there was a 90-day follow-up that, although clinical, may not be able to identify long-term complications of dysphagia. Moreover, self-reporting might be required to determine hospital readmissions, and thus, a recall bias is possible. Another limitation is that we did not stratify dysphagia into severity grades (mild, moderate, and severe) beyond the total EAT-10 score; therefore, regression analysis based on dysphagia grades could not be conducted. Furthermore, the study did not collect or report mortality data during the 90-day follow-up, which might have provided additional insight into the risks associated with dysphagia and aspiration pneumonia. Additionally, we did not perform separate univariate analyses for individual predictors before conducting the multivariate logistic regression. Consequently, variables with weak or nonsignificant associations in isolation were assessed only within the multivariate model. Future studies may consider univariate screening to further clarify independent risk factors. Finally, the EAT-10 has not been demonstrated to replace alternative objective screening methodologies of swallowing, such as VFSS or FEES, which may be more diagnostic.

Recommendations for future research

Future studies should adopt a longitudinal design with long follow-up periods to ascertain the long-term impact of dysphagia on health outcomes. Additional objective swallowing tests, such as the EAT-10, would provide a more detailed image. Furthermore, interventional studies on the effectiveness of early management methods for dysphagia, such as swallowing therapy, dietary modification, and multidisciplinary discharge planning, are necessary to determine the most effective strategies for reducing the number of victims of aspiration pneumonia and the rate of readmission. The research should be extended to other areas in Pakistan, and the results should be compared across various healthcare environments to enhance external validity and gather more information about healthcare planning in Pakistan.

## Conclusions

This study indicates that predischarge dysphagia, as assessed by EAT-10, is a strong predictor of 90-day aspiration pneumonia and 90-day hospital readmission. Age and comorbidity also contribute to the increase of these risks, as well as a history of pneumonia and prolonged hospitalization. Dysphagia screening before discharge can also help identify patients at risk and allow clinicians to take prompt measures, enabling such patients to avoid potential complications. EAT-10 can be introduced into a discharge process as a possible cost-effective approach to enhancing patient safety and continuity of care, particularly in resource-constrained healthcare systems.

## Appendices

**Table 8:** Demographic information questionnaire

Items	Responses
Age	-
Gender	-
Marital status	-
Educational level	-
Occupation	-
Primary diagnosis (cause of admission)	-
Feeding status at discharge	-
Comorbidities (tick all that apply)	-
Smoking status	-
History of pneumonia in the past 12 months	-
Length of hospital stay (current admission)	-

**Table 9:** 90-day follow-up section questionnaire

Items	Response
Since your hospital discharge, have you been diagnosed with pneumonia?	-
Have you been readmitted to the hospital within 90 days after discharge?	-

**Table 10:** Eating Assessment Tool (EAT-10) Formal permission for the use of the Eating Assessment Tool (EAT-10) was obtained from Mapi Research Trust (Special Terms No. 121857). Credit: Belafsky et al. (2008) [16]

Items	Responses
My swallowing problem has caused me to lose weight.	-
My swallowing problem interferes with my ability to go out for meals.	-
Swallowing liquids takes extra effort.	-
Swallowing solids takes extra effort.	-
Swallowing pills takes extra effort.	-
Swallowing is painful.	-
The pleasure of eating is affected by my swallowing.	-
When I swallow food sticks in my throat.	-
I cough when I eat.	-
Swallowing is stressful.	-
